# Reply to Romano *et al.*

**DOI:** 10.1093/icvts/ivac172

**Published:** 2022-07-13

**Authors:** Gowthanan Santhirakumaran, Ali Abbasi, Mohammad Shah, Ian Hunt

**Affiliations:** Cardiothoracic Surgery, St George’s University Hospital, London, UK; Radiology Department, University College Hospital, London, UK; Radiology Department, St George’s University Hospital, London, UK; Cardiothoracic Surgery, St George’s University Hospital, London, UK

**Keywords:** Slipping rib syndrome, Dynamic ultrasound of chest wall, Chest wall conditions

We commend Romano *et al.* [1] in detailing an interesting sign for slipping rib syndrome (SRS). As widely understood, SRS is commonly a clinical diagnosis. However, as illustrated by this case series, the emergence of ultrasound has allowed clinicians to identify SRS more confidently, adding to a positive hook manoeuvre [2]. The associated atrophy of rectus abdominis muscle in this case series implicates it because of nerve entrapment and also further worsens the costal cartilage instability. However, this finding could also be used to improve treatment of SRS. Surgical intervention has been identified as the mainstay for SRS, however, with this finding, physiotherapy can be key prior to consideration of surgery, as increasing core strength can improve costal arch stability and in turn, patient symptoms [3]. However, this can be proven to be difficult as the very movements in the exercises can precipitate pain, and therefore, surgical resection has maintained its gold standard.

In our experience, we have found ultrasound to be a crucial investigative tool in diagnosing and managing SRS. Ultrasound imaging provides a useful static image of muscle hypotrophy as detailed excellently in this SRS case series, however, the dynamic use of ultrasound can be argued to be a more important investigation in diagnosing SRS. This has already been reported by thoracic surgeons and musculoskeletal radiologists who treat SRS as vital to their practice with promising results. We have implemented a protocol for the use of dynamic ultrasound of the chest wall in our unit as a diagnostic tool in patients suspected of SRS. By having patients reproduce the movements causing pain, dynamic ultrasound of the chest wall performed by a specialist musculoskeletal radiologist can delineate anatomy and illustrate the excursion of the costal cartilage to the rib above, causing nerve entrapment and in turn, pain in real time [4]. This not only reduces the room for interpretation as commonly found with the hook manoeuvre but also adds important information to the surgeon in preoperative planning. Dynamic ultrasound of the chest wall can illustrate exactly which rib tip’s hypermobility is causing the pain and can aid identification for targeted diagnostic/therapeutic intercostal nerve blocks as well as costal cartilage resection [4–6].

In conclusion, the case series of Romano *et al.* provides new knowledge for a relatively underdiagnosed condition and illustrate the increasing importance of ultrasound imaging, for which we point the emergence of its dynamic use in SRS.
